# 2-{[(4-Methoxy­phen­yl)dimethyl­silyl]meth­yl}isoindoline-1,3-dione

**DOI:** 10.1107/S1600536809054129

**Published:** 2009-12-24

**Authors:** Ilia A. Guzei, Lara C. Spencer, Uzma I. Zakai

**Affiliations:** a Department of Chemistry, University of Wisconsin–Madison, 1101 University Ave, Madison, Wisconsin 53706, USA

## Abstract

In the course of our studies of silicon-containing anti­cancer compounds, the title compound, C_18_H_19_NO_3_Si, was synthesized. The mol­ecular geometry including bond distances and angles involving the Si atoms are typical. Torsion angles associated with the isoindoline ring and the silyl group [C—N—C_methyl­ene_—Si = 90.5 (2) and −93.1 (2)°] indicate that there is no inter­action between the O and Si atoms despite silicon’s high affinity for oxygen.

## Related literature

For literature related to drug design see: Bains & Tacke (2003 ▶); Bikzhanova *et al.* (2007 ▶); Franz (2007 ▶); Franz *et al.* (2007 ▶); Gately & West (2007 ▶); Guzei, Spencer, Zakai & Lynch (2010 ▶); Guzei, Spencer & Zakai (2010 ▶); Latxague & Leger (2004 ▶); Lee *et al.* (1993 ▶, 1996 ▶); Murai *et al.* (1998 ▶); Showell & Mills (2003 ▶); Tacke & Zilch (1986 ▶); Tsuge *et al.* (1985 ▶); Yoon *et al.* (1991 ▶, 1992 ▶, 1997 ▶). For a description of the Cambridge Structural Database, see: Allen (2002 ▶). Bond distances and angles were confirmed to be typical by a *Mogul* structural check (Bruno *et al.*, 2004 ▶).
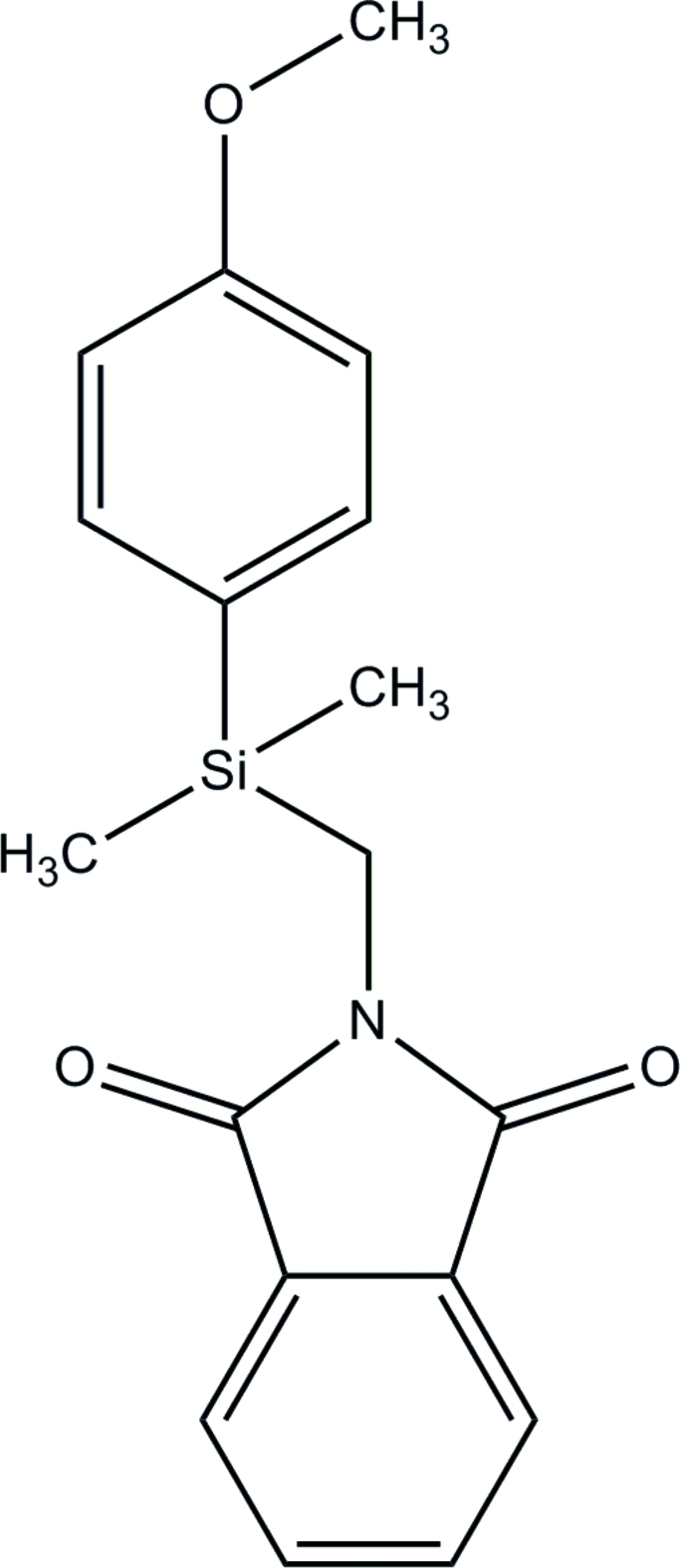


## Experimental

### 

#### Crystal data

C_18_H_19_NO_3_Si
*M* *_r_* = 325.43
Monoclinic, 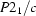

*a* = 10.2713 (16) Å
*b* = 14.061 (3) Å
*c* = 12.069 (2) Å
β = 103.355 (6)°
*V* = 1695.9 (5) Å^3^
*Z* = 4
Mo *K*α radiation
μ = 0.15 mm^−1^
*T* = 300 K
0.50 × 0.40 × 0.23 mm

#### Data collection

Bruker SMART X2S diffractometer
Absorption correction: multi-scan (*SADABS*; Bruker, 2009 ▶) *T*_min_ = 0.928, *T*_max_ = 0.966
11394 measured reflections
3197 independent reflections
2338 reflections with *I* > 2σ(*I*)
*R*_int_ = 0.042

#### Refinement

*R*[*F* ^2^ > 2σ(*F* ^2^)] = 0.046
*wR*(*F* ^2^) = 0.131
*S* = 0.96
3197 reflections
211 parameters
H-atom parameters constrained
Δρ_max_ = 0.21 e Å^−3^
Δρ_min_ = −0.26 e Å^−3^

### 

Data collection: *APEX2* and *GIS* (Bruker, 2009 ▶); cell refinement: *SAINT* (Bruker, 2009 ▶); data reduction: *SAINT*; program(s) used to solve structure: *SHELXTL* (Sheldrick, 2008 ▶); program(s) used to refine structure: *SHELXTL*, *OLEX2* (Dolomanov *et al.*, 2009 ▶) and *FCF_filter* (Guzei, 2007 ▶); molecular graphics: *SHELXTL*; software used to prepare material for publication: *SHELXTL*, *modiCIFer* (Guzei, 2007 ▶) and *publCIF* (Westrip, 2010 ▶).

## Supplementary Material

Crystal structure: contains datablocks global, I. DOI: 10.1107/S1600536809054129/zs2023sup1.cif

Structure factors: contains datablocks I. DOI: 10.1107/S1600536809054129/zs2023Isup2.hkl

Additional supplementary materials:  crystallographic information; 3D view; checkCIF report

## Figures and Tables

**Fig. 1.**
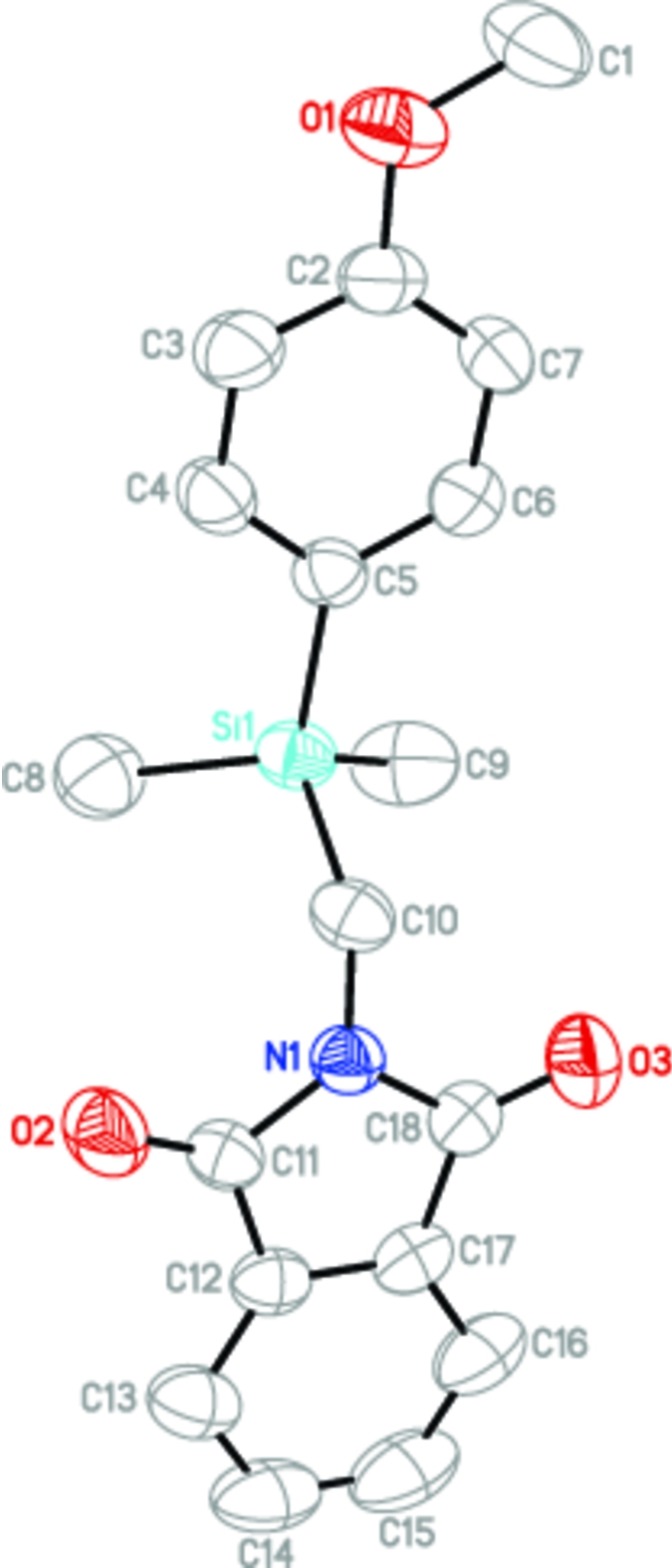
Molecular structure of (I). The thermal ellipsoids are shown at 50% probability level.

## supplementary crystallographic information

### Comment

Sila phthalimides are important intermediates in photochemistry (Lee *et al*., 1993, 1996; Yoon *et al.*, 1997, 1992, 1991) and organic synthesis (Bikzhanova *et al.*, 2007; Tsuge *et al.*, 1985). We have used such compounds for the synthesis of selectively substituted sila amines (Bikzhanova *et al.*, 2007) in order to identify biologically active organosilicon compounds. Organosilicon chemistry is a growing method of expanding chemical diversity (Franz, 2007; Franz *et al.*, 2007; Tacke & Zilch, 1986; Showell & Mills, 2003), and it constitutes a powerful method of enhancing pharmacological properties in drug design (Bains & Tacke, 2003). In the course of our studies of silicon-containing anti-cancer compounds the title compound, (I), was synthesized and its structure is reported here.

The bond distances and angles of (I) are typical as confirmed by the *Mogul* structural check (Bruno *et al.*, 2004), and agree well with those for 2-(3-(methyldiphenylsilyl)propyl)isoindoline-1,3-dione (Guzei, Spencer, Zakai & Lynch, 2010) and 2-(2-(trimethylsilyl)ethyl)isoindoline-1,3-dione (Guzei, Spencer & Zakai, 2010). Specifically, the average Si—C distances of 1.868 (19) Å for compound (I) are statistically similar to the 1.859 (6) Å average for six related compounds in the Cambridge Structural Database (Version 1.11, September 2009 release; Allen, 2002). The Si atom has a distorted tetrahedral geometry with angles ranging from 107.86 (10)° to 110.81 (13)°. Torsion angles C(11/18)-N1-C10-Si1 involving the silyl group are 90.5 (2) and -93.1 (2)°, similar to those in the related compound 6-(phthalimidomethyl(dimethyl)silyl)hexan-1-ol (Latxague & Leger, 2004). This is an indication that there is no interaction between the carbonyl oxygen and the silicon atom despite silicon's high affinity for oxygen (Murai *et al.*, 1998).

The phthalate entity is planar within 0.0084 Å, and the methoxyphenyl group within 0.0044 Å. These groups are nearly parallel forming a 4.61 (8)° angle between their planes. There is one non-classical intermolecular interaction C4–H4···O2 with a C···O distance of 3.410 (3) Å and a C—H ···O angle of 134°. This weak interaction helps link the molecules of (I) into a three-dimensional framework.

### Experimental

The protocol described by Tsuge and co-workers (Tsuge *et al.*, 1985) was adopted. The required amount of potassium phthalimide (4.79 g, 25.88 mmol, 1.1 equiv) was placed into a 100 ml round-bottom flask, which was then sealed and flushed with nitrogen three times. Dry DMF (36 ml) was syringed into the flask followed by the addition of 4-methoxybenzylchloride (5.05 g, 23.53 mmol, 1 equiv.) The reaction was heated at 60°C for 6 h and the resulting mixture was then allowed to cool to room temperature. This slurry was poured onto a minimum quantity of water and extracted 3–5 times with ethyl ether. The organic extracts were subsequently collected, dried with magnesium sulfate, and filtered. The filtrate was mixed with silica gel and evaporated under reduced pressure to afford a powder of silica gel. This powder was loaded onto a dry-packed silica gel column and eluted using a gradient column. The fractions of interest were mobilized using a 8:2 hexane:ethyl acetate mixture but did not completely elute until a 1:1 hex:EtOAc mixture was employed. The fractions were then combined to afford the desired compound. Further recrystallization from dichloromethane afforded cream colored crystals (5.35 g, 16.44 mmol, 70% yield) for X-ray crystallography. Manipulation of air and moisture sensitive compounds was performed using standard high-vacuum line techniques. All solvents and reagents were obtained from Aldrich. 4-Methoxybenzylchloride was purchased from Acros Organics. ^1^H NMR spectra were obtained on a Varian Unity 500 spectrometer, ^13^C {H} NMR spectra were obtained on a Varian 500 spectrometer operating at 125 MHz, ^29^Si {H} NMR spectra were obtained on a Varian Unity spectrometer operating at 99 MHz. Mass spectra were determined on a Waters (Micromass) AutoSpec mass spectrometer. Melting points were determined on a Mel-Temp Laboratory Device. mp 75–77°C; ^1^H NMR (500 MHz, CDCl_3_) δ 0.36 (s, 6H, CH_3_), 3.35 (s, 2H, CH_2_), 3.77 (s, 3H, OMe), 6.87 (m, 2H, ArH), 7.47 (m, 2H, ArH), 7.64 (dd, *J*=5.45, 3.01 Hz, 2H, ArH), 7.75 (dd, *J*=5.38, 3.07 Hz, 2H, ArH); ^13^C NMR (125 MHz, CDCl_3_) δ -2.9 (SiCH_3_), 28.8 (CH_2_), 55.0 (OMe), 113.6 (CH), 122.8 (CH), 127.1 (CH), 132.2 (CH), 133.5 (CH), 135.2 (CH), 160.7 (CH), 168.4 (CH); ^29^Si NMR (99 MHz, CDCl_3_) δ -3.17 (SiMe_2_PhOMe); MS (EI^+^) *m/z* (*rel*. intensity %) 324 (*M*-1, 9), 310 (M—Me, 100), 218 (56), 165 (75); HRMS (EI^+^): calcd. for C_18_H_19_NO_3_Si (*M*^+^) 325.1129, found (M—Me)^+^ 310.0894.

### Refinement

All H-atoms were placed in idealized locations and refined as riding with appropriate thermal displacement coefficients *U*_iso_(H) = 1.2 or 1.5 times *U*_eq_(bearing atom). The data were collected at room temperature on a Bruker SMART X2S diffractometer in the automated mode and manually processed thereafter.

### Crystal data

**Table:**

C_18_H_19_NO_3_Si	*F*(000) = 688
*M**_r_* = 325.43	*D*_x_ = 1.275 Mg m^−^^3^
Monoclinic, *P*2_1_/*c*	Mo *K*α radiation, λ = 0.71073 Å
Hall symbol: -P 2ybc	Cell parameters from 3201 reflections
*a* = 10.2713 (16) Å	θ = 2.3–24.8°
*b* = 14.061 (3) Å	µ = 0.15 mm^−^^1^
*c* = 12.069 (2) Å	*T* = 300 K
β = 103.355 (6)°	Block, colourless
*V* = 1695.9 (5) Å^3^	0.50 × 0.40 × 0.23 mm
*Z* = 4	

### Data collection

**Table:**

Bruker SMART X2S diffractometer	3197 independent reflections
Radiation source: micro-focus sealed tube	2338 reflections with *I* > 2σ(*I*)
doubly curved silicon crystal	*R*_int_ = 0.042
ω scans	θ_max_ = 25.7°, θ_min_ = 2.5°
Absorption correction: multi-scan (*SADABS*; Bruker, 2009)	*h* = −12→12
*T*_min_ = 0.928, *T*_max_ = 0.966	*k* = −17→17
11394 measured reflections	*l* = −11→14

### Refinement

**Table:**

Refinement on *F*^2^	Primary atom site location: structure-invariant direct methods
Least-squares matrix: full	Secondary atom site location: difference Fourier map
*R*[*F*^2^ > 2σ(*F*^2^)] = 0.046	Hydrogen site location: inferred from neighbouring sites
*wR*(*F*^2^) = 0.131	H-atom parameters constrained
*S* = 0.96	*w* = 1/[σ^2^(*F*_o_^2^) + (0.0812*P*)^2^ + 0.1363*P*] where *P* = (*F*_o_^2^ + 2*F*_c_^2^)/3
3197 reflections	(Δ/σ)_max_ < 0.001
211 parameters	Δρ_max_ = 0.21 e Å^−^^3^
0 restraints	Δρ_min_ = −0.26 e Å^−^^3^

### Special details

**Table:**

Geometry. All e.s.d.'s (except the e.s.d. in the dihedral angle between two l.s. planes) are estimated using the full covariance matrix. The cell e.s.d.'s are taken into account individually in the estimation of e.s.d.'s in distances, angles and torsion angles; correlations between e.s.d.'s in cell parameters are only used when they are defined by crystal symmetry. An approximate (isotropic) treatment of cell e.s.d.'s is used for estimating e.s.d.'s involving l.s. planes.
Refinement. Refinement of *F*^2^ against ALL reflections. The weighted *R*-factor *wR* and goodness of fit *S* are based on *F*^2^, conventional *R*-factors *R* are based on *F*, with *F* set to zero for negative *F*^2^. The threshold expression of *F*^2^ > σ(*F*^2^) is used only for calculating *R*-factors(gt) *etc*. and is not relevant to the choice of reflections for refinement. *R*-factors based on *F*^2^ are statistically about twice as large as those based on *F*, and *R*- factors based on ALL data will be even larger.

### Fractional atomic coordinates and isotropic or equivalent isotropic displacement parameters (Å^2^)

**Table:**

	*x*	*y*	*z*	*U*_iso_*/*U*_eq_	
Si1	−0.00370 (6)	0.19378 (4)	0.82075 (5)	0.0462 (2)	
O1	−0.34928 (17)	0.48107 (11)	0.99174 (16)	0.0762 (5)	
O2	0.14405 (16)	−0.05076 (11)	0.93970 (14)	0.0674 (5)	
O3	0.38001 (16)	0.20812 (11)	0.87061 (17)	0.0779 (5)	
N1	0.23597 (15)	0.09334 (11)	0.90879 (13)	0.0439 (4)	
C1	−0.3261 (3)	0.57979 (18)	0.9894 (3)	0.0880 (9)	
H1A	−0.2364	0.5935	1.0307	0.132*	
H1B	−0.3884	0.6127	1.0240	0.132*	
H1C	−0.3377	0.6004	0.9119	0.132*	
C2	−0.2647 (2)	0.42125 (15)	0.95212 (18)	0.0518 (5)	
C3	−0.2964 (2)	0.32606 (16)	0.9526 (2)	0.0603 (6)	
H3	−0.3712	0.3068	0.9779	0.072*	
C4	−0.2182 (2)	0.25971 (15)	0.91604 (18)	0.0532 (5)	
H4	−0.2403	0.1958	0.9189	0.064*	
C5	−0.10617 (18)	0.28429 (13)	0.87447 (16)	0.0426 (5)	
C6	−0.0790 (2)	0.38081 (15)	0.87433 (18)	0.0521 (5)	
H6	−0.0060	0.4007	0.8470	0.063*	
C7	−0.1555 (2)	0.44937 (15)	0.91292 (19)	0.0552 (6)	
H7	−0.1330	0.5134	0.9122	0.066*	
C8	−0.1060 (2)	0.08665 (17)	0.7693 (2)	0.0682 (7)	
H8A	−0.1350	0.0579	0.8316	0.102*	
H8B	−0.0531	0.0419	0.7385	0.102*	
H8C	−0.1827	0.1048	0.7112	0.102*	
C9	0.0661 (3)	0.2458 (2)	0.7055 (2)	0.0755 (7)	
H9A	−0.0049	0.2730	0.6484	0.113*	
H9B	0.1098	0.1969	0.6719	0.113*	
H9C	0.1297	0.2944	0.7367	0.113*	
C10	0.1385 (2)	0.15592 (15)	0.94299 (17)	0.0494 (5)	
H10A	0.1844	0.2123	0.9785	0.059*	
H10B	0.1014	0.1235	0.9996	0.059*	
C11	0.2303 (2)	−0.00539 (14)	0.91102 (16)	0.0449 (5)	
C12	0.34907 (19)	−0.03966 (14)	0.87220 (16)	0.0466 (5)	
C13	0.3901 (2)	−0.13083 (17)	0.85585 (19)	0.0637 (6)	
H13	0.3418	−0.1836	0.8699	0.076*	
C14	0.5067 (3)	−0.1404 (2)	0.8175 (2)	0.0792 (8)	
H14	0.5375	−0.2009	0.8055	0.095*	
C15	0.5775 (3)	−0.0621 (2)	0.7968 (2)	0.0838 (9)	
H15	0.6555	−0.0710	0.7715	0.101*	
C16	0.5365 (2)	0.0289 (2)	0.8124 (2)	0.0713 (7)	
H16	0.5847	0.0815	0.7977	0.086*	
C17	0.4204 (2)	0.03886 (15)	0.85097 (17)	0.0498 (5)	
C18	0.34950 (19)	0.12526 (16)	0.87585 (17)	0.0515 (5)	

### Atomic displacement parameters (Å^2^)

**Table:**

	*U*^11^	*U*^22^	*U*^33^	*U*^12^	*U*^13^	*U*^23^
Si1	0.0503 (4)	0.0469 (3)	0.0444 (3)	0.0088 (2)	0.0174 (3)	0.0027 (2)
O1	0.0752 (11)	0.0601 (10)	0.1004 (13)	0.0179 (8)	0.0350 (10)	−0.0101 (9)
O2	0.0723 (11)	0.0521 (9)	0.0872 (12)	0.0016 (8)	0.0377 (9)	0.0162 (8)
O3	0.0658 (11)	0.0540 (11)	0.1174 (15)	−0.0131 (8)	0.0284 (10)	0.0015 (9)
N1	0.0430 (9)	0.0418 (9)	0.0487 (9)	0.0035 (7)	0.0143 (7)	0.0013 (7)
C1	0.0881 (19)	0.0571 (16)	0.116 (2)	0.0182 (14)	0.0166 (17)	−0.0252 (15)
C2	0.0478 (12)	0.0503 (12)	0.0567 (13)	0.0113 (10)	0.0112 (10)	−0.0017 (10)
C3	0.0551 (13)	0.0580 (14)	0.0758 (16)	0.0070 (11)	0.0316 (12)	0.0077 (11)
C4	0.0581 (13)	0.0403 (11)	0.0665 (13)	0.0034 (10)	0.0253 (11)	0.0063 (10)
C5	0.0425 (11)	0.0424 (11)	0.0420 (10)	0.0042 (8)	0.0080 (8)	0.0054 (8)
C6	0.0450 (11)	0.0482 (12)	0.0646 (13)	−0.0004 (9)	0.0156 (10)	0.0031 (10)
C7	0.0550 (13)	0.0365 (11)	0.0704 (14)	0.0007 (9)	0.0069 (11)	−0.0012 (10)
C8	0.0661 (15)	0.0660 (15)	0.0713 (15)	0.0044 (12)	0.0136 (12)	−0.0197 (12)
C9	0.0809 (17)	0.0925 (19)	0.0626 (15)	0.0215 (15)	0.0363 (13)	0.0217 (14)
C10	0.0564 (12)	0.0448 (11)	0.0501 (12)	0.0086 (9)	0.0187 (10)	−0.0041 (9)
C11	0.0504 (12)	0.0418 (11)	0.0430 (11)	0.0051 (9)	0.0116 (9)	0.0052 (9)
C12	0.0459 (11)	0.0519 (12)	0.0389 (11)	0.0082 (9)	0.0033 (9)	−0.0011 (9)
C13	0.0690 (15)	0.0558 (14)	0.0628 (14)	0.0159 (12)	0.0077 (12)	−0.0073 (11)
C14	0.0711 (17)	0.085 (2)	0.0755 (17)	0.0336 (15)	0.0050 (14)	−0.0252 (15)
C15	0.0519 (15)	0.119 (3)	0.0816 (18)	0.0232 (17)	0.0187 (13)	−0.0206 (18)
C16	0.0421 (12)	0.095 (2)	0.0787 (17)	0.0064 (12)	0.0175 (11)	−0.0080 (14)
C17	0.0388 (11)	0.0629 (14)	0.0456 (11)	0.0041 (9)	0.0053 (9)	−0.0016 (10)
C18	0.0440 (11)	0.0517 (13)	0.0569 (13)	−0.0012 (10)	0.0080 (9)	0.0019 (10)

### Geometric parameters (Å, °)

**Table:**

Si1—C9	1.856 (2)	C6—H6	0.9300
Si1—C8	1.859 (2)	C7—H7	0.9300
Si1—C5	1.8609 (19)	C8—H8A	0.9600
Si1—C10	1.897 (2)	C8—H8B	0.9600
O1—C2	1.372 (2)	C8—H8C	0.9600
O1—C1	1.410 (3)	C9—H9A	0.9600
O2—C11	1.205 (2)	C9—H9B	0.9600
O3—C18	1.212 (2)	C9—H9C	0.9600
N1—C11	1.390 (3)	C10—H10A	0.9700
N1—C18	1.390 (2)	C10—H10B	0.9700
N1—C10	1.462 (2)	C11—C12	1.485 (3)
C1—H1A	0.9600	C12—C13	1.378 (3)
C1—H1B	0.9600	C12—C17	1.381 (3)
C1—H1C	0.9600	C13—C14	1.386 (3)
C2—C7	1.372 (3)	C13—H13	0.9300
C2—C3	1.378 (3)	C14—C15	1.374 (4)
C3—C4	1.369 (3)	C14—H14	0.9300
C3—H3	0.9300	C15—C16	1.373 (4)
C4—C5	1.400 (3)	C15—H15	0.9300
C4—H4	0.9300	C16—C17	1.383 (3)
C5—C6	1.386 (3)	C16—H16	0.9300
C6—C7	1.390 (3)	C17—C18	1.482 (3)
			
C9—Si1—C8	110.81 (13)	H8A—C8—H8C	109.5
C9—Si1—C5	109.82 (10)	H8B—C8—H8C	109.5
C8—Si1—C5	110.42 (10)	Si1—C9—H9A	109.5
C9—Si1—C10	109.38 (11)	Si1—C9—H9B	109.5
C8—Si1—C10	107.86 (10)	H9A—C9—H9B	109.5
C5—Si1—C10	108.48 (8)	Si1—C9—H9C	109.5
C2—O1—C1	118.2 (2)	H9A—C9—H9C	109.5
C11—N1—C18	111.69 (16)	H9B—C9—H9C	109.5
C11—N1—C10	124.13 (16)	N1—C10—Si1	113.82 (13)
C18—N1—C10	124.10 (17)	N1—C10—H10A	108.8
O1—C1—H1A	109.5	Si1—C10—H10A	108.8
O1—C1—H1B	109.5	N1—C10—H10B	108.8
H1A—C1—H1B	109.5	Si1—C10—H10B	108.8
O1—C1—H1C	109.5	H10A—C10—H10B	107.7
H1A—C1—H1C	109.5	O2—C11—N1	124.82 (18)
H1B—C1—H1C	109.5	O2—C11—C12	129.12 (19)
O1—C2—C7	125.3 (2)	N1—C11—C12	106.06 (17)
O1—C2—C3	115.12 (19)	C13—C12—C17	121.6 (2)
C7—C2—C3	119.63 (19)	C13—C12—C11	130.4 (2)
C4—C3—C2	120.2 (2)	C17—C12—C11	108.00 (17)
C4—C3—H3	119.9	C12—C13—C14	117.0 (2)
C2—C3—H3	119.9	C12—C13—H13	121.5
C3—C4—C5	122.63 (19)	C14—C13—H13	121.5
C3—C4—H4	118.7	C15—C14—C13	121.1 (2)
C5—C4—H4	118.7	C15—C14—H14	119.4
C6—C5—C4	115.11 (18)	C13—C14—H14	119.4
C6—C5—Si1	122.64 (15)	C16—C15—C14	122.0 (2)
C4—C5—Si1	122.24 (15)	C16—C15—H15	119.0
C5—C6—C7	123.3 (2)	C14—C15—H15	119.0
C5—C6—H6	118.3	C15—C16—C17	117.1 (3)
C7—C6—H6	118.3	C15—C16—H16	121.4
C2—C7—C6	119.06 (19)	C17—C16—H16	121.4
C2—C7—H7	120.5	C12—C17—C16	121.1 (2)
C6—C7—H7	120.5	C12—C17—C18	108.11 (18)
Si1—C8—H8A	109.5	C16—C17—C18	130.8 (2)
Si1—C8—H8B	109.5	O3—C18—N1	124.7 (2)
H8A—C8—H8B	109.5	O3—C18—C17	129.2 (2)
Si1—C8—H8C	109.5	N1—C18—C17	106.09 (18)
			
C1—O1—C2—C7	2.1 (3)	C18—N1—C11—C12	−2.0 (2)
C1—O1—C2—C3	−177.5 (2)	C10—N1—C11—C12	−178.81 (15)
O1—C2—C3—C4	−179.3 (2)	O2—C11—C12—C13	1.9 (4)
C7—C2—C3—C4	1.1 (3)	N1—C11—C12—C13	−178.2 (2)
C2—C3—C4—C5	−1.6 (3)	O2—C11—C12—C17	−178.8 (2)
C3—C4—C5—C6	0.8 (3)	N1—C11—C12—C17	1.1 (2)
C3—C4—C5—Si1	−178.04 (17)	C17—C12—C13—C14	0.2 (3)
C9—Si1—C5—C6	−30.5 (2)	C11—C12—C13—C14	179.45 (19)
C8—Si1—C5—C6	−153.00 (17)	C12—C13—C14—C15	0.0 (4)
C10—Si1—C5—C6	88.99 (18)	C13—C14—C15—C16	−0.3 (4)
C9—Si1—C5—C4	148.23 (18)	C14—C15—C16—C17	0.4 (4)
C8—Si1—C5—C4	25.73 (19)	C13—C12—C17—C16	−0.1 (3)
C10—Si1—C5—C4	−92.27 (18)	C11—C12—C17—C16	−179.46 (18)
C4—C5—C6—C7	0.4 (3)	C13—C12—C17—C18	179.50 (18)
Si1—C5—C6—C7	179.23 (16)	C11—C12—C17—C18	0.1 (2)
O1—C2—C7—C6	−179.48 (19)	C15—C16—C17—C12	−0.3 (3)
C3—C2—C7—C6	0.0 (3)	C15—C16—C17—C18	−179.7 (2)
C5—C6—C7—C2	−0.8 (3)	C11—N1—C18—O3	−177.6 (2)
C11—N1—C10—Si1	−93.1 (2)	C10—N1—C18—O3	−0.8 (3)
C18—N1—C10—Si1	90.5 (2)	C11—N1—C18—C17	2.1 (2)
C9—Si1—C10—N1	−53.22 (18)	C10—N1—C18—C17	178.88 (16)
C8—Si1—C10—N1	67.39 (17)	C12—C17—C18—O3	178.3 (2)
C5—Si1—C10—N1	−172.99 (14)	C16—C17—C18—O3	−2.2 (4)
C18—N1—C11—O2	177.87 (19)	C12—C17—C18—N1	−1.3 (2)
C10—N1—C11—O2	1.1 (3)	C16—C17—C18—N1	178.2 (2)
